# Women’s contribution in understanding how topoisomerases, supercoiling, and transcription control genome organization

**DOI:** 10.3389/fmolb.2023.1155825

**Published:** 2023-03-27

**Authors:** Laura Martin, Maria Victoria Neguembor, Maria Pia Cosma

**Affiliations:** ^1^ Centre for Genomic Regulation (CRG), The Barcelona Institute of Science and Technology, Barcelona, Spain; ^2^ Technical Contact, Guangzhou, China; ^3^ ICREA, Barcelona, Spain; ^4^ Medical Research Institute, Guangdong Provincial People’s Hospital (Guangdong Academy of Medical Sciences), Southern Medical University, Guangzhou, China; ^5^ Universitat Pompeu Fabra (UPF), Barcelona, Spain; ^6^ Lead Contact, Guangzhou, China

**Keywords:** genome organization, supercoiling, topoisomerases, transcription, women

## Abstract

One of the biggest paradoxes in biology is that human genome is roughly 2 m long, while the nucleus containing it is almost one million times smaller. To fit into the nucleus, DNA twists, bends and folds into several hierarchical levels of compaction. Still, DNA has to maintain a high degree of accessibility to be readily replicated and transcribed by proteins. How compaction and accessibility co-exist functionally in human cells is still a matter of debate. Here, we discuss how the torsional stress of the DNA helix acts as a buffer, regulating both chromatin compaction and accessibility. We will focus on chromatin supercoiling and on the emerging role of topoisomerases as pivotal regulators of genome organization. We will mainly highlight the major breakthrough studies led by women, with the intention of celebrating the work of this group that remains a minority within the scientific community.

## Introduction

### Multiple levels of organization shape the genome’s structure

In human somatic nuclei, genetic information is stored into 46 molecules of DNA, called chromosomes, that differ in length and base sequence (Tjio and Levan, 1956). During the different phases of the cell cycle, chromosomes undergo dramatic reorganization, transitioning from an extremely condensed form during mitosis to a mostly decondensed form in interphase (Bolzer et al., 2005).

**FIGURE 1:**
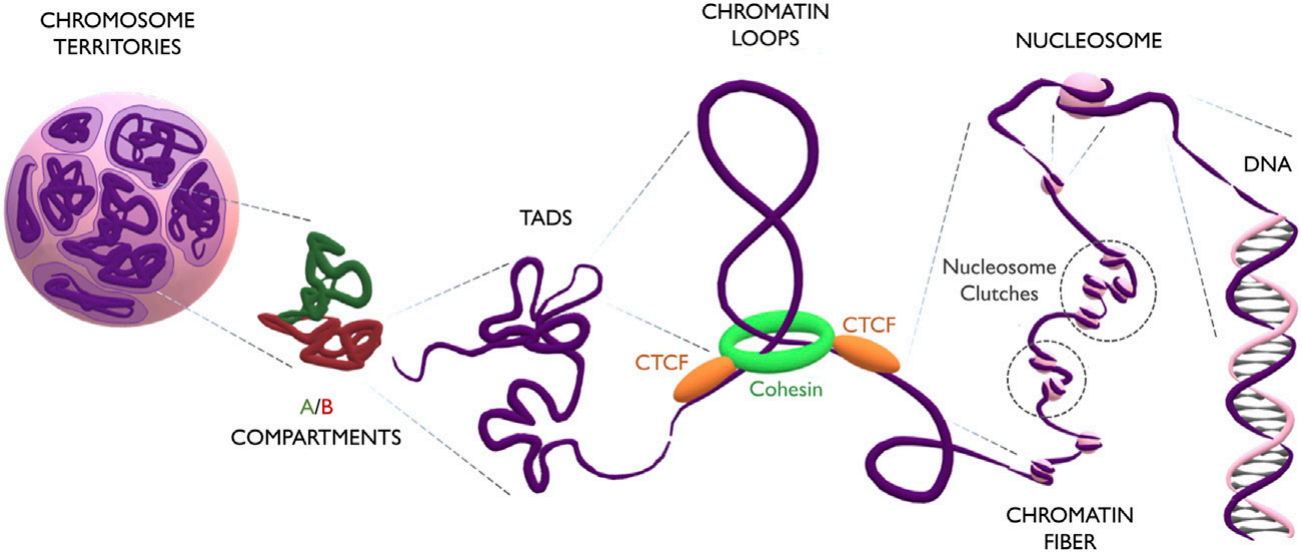
The multilayered organization of DNA. Schematic representation of genome folding at multiple scales. Chromosomes (in purple) occupy discrete areas called chromosome territories. Inside each chromosome, active A compartments (in green) segregate from inactive B compartments (in red). Compartments organize into big TADs (∼Mb) and smaller loops (∼kb). Loops form by the extrusion of chromatin through the cohesin ring (in green) which finally stalls at CTCF anchoring points (in orange). The loop is constituted by the chromatin fiber, which contains heterogeneous groups of nucleosome clutches interspersed along the genome (highlighted in dashed circles). Nucleosomes (pink spheres) are formed by a histone core, around which the DNA helix is wrapped.

During interphase, single chromosomes occupy distinct parts of the nuclear volume, called chromosome territories (Dietzel et al., 1998). Inside each chromosome, more transcriptionally active regions segregate from repressed ones, forming respectively A and B compartments (Rao et al., 2014). On a smaller scale, compartments are further organized into domains of highly interacting chromatin, which exhibit distinct characteristics depending on their association with the nuclear membrane, nucleolus, or nucleoplasm. These domains are referred to Lamina Associated Domains (LADs), Nucleolar Associated Domains (NADs), and Topologically Associated Domains (TADs), respectively (Guelen et al., 2008; Németh et al., 2010; Dixon et al., 2012). Notably, the size of these domains is variable and still controversial, as its measurement is directly affected by the binning and resolution of Hi-C maps: while initially domains were reported to span several megabases, high-resolution contact maps have recently indicated domains of only few kilobases, also known as “chromatin loops” (Rowley and Corces, 2018). Loops form through the extrusion of chromatin by a ring-shape complex of cohesin proteins (RAD21, SMC1A, SMC3 and SA1/2) (Michaelis et al., 1997; Losada et al., 2000; Sumara et al., 2000; Wendt et al., 2008). Extrusion proceeds until cohesin stalls when met by two CTCF proteins, which act as insulators (Alipour and Marko, 2012). CTCFs binds DNA at CTCF-binding motifs which, being asymmetric, can have two different orientations (5′-->3 or 3′-->5′). Interestingly, convergent orientation of CTCF motifs is important for the formation of the loop structure (Sanborn et al., 2015; Fudenberg and, Imakaev, 2016), suggesting that stalling of cohesin happens exclusively through the C-terminal domain of CTCF (Xiao et al., 2011). The chromatin loop is interspersed with nucleosomes, which are complexes of 8 histone proteins (pairs of H2A, H2B, H3 and H4) forming a core around which the DNA molecule is wrapped (Kornberg, 1974). The linker histone H1 binds DNA entering and exiting the nucleosome, and the local enrichment of H1 plays an important role in epigenetic regulation, DNA replication, genome stability, and chromatin organization (Simpson, 1978; Fyodorov et al., 2018). Nucleosomes do not group together into a rigid and symmetric configuration, as believed for many decades (Finch and Klug, 1976), but form instead a pearl-necklace structure of clutches of different densities, depending on the epigenetic and transcriptional status of the chromatin region or on the potency of the cell (Olins and Olins, 1974; Ricci et al., 2015). Finally, DNA is a helix of two antiparallel filaments composed of nucleotides (Franklin and Gosling, 1953; Watson and Crick, 1953; Wilkins et al., 1953). Every nucleotide consists of a 2′-deoxyribose sugar, a phosphate group and one of the four nitrogenous bases (Adenine, Guanine, Thymine or Cytosine). The DNA sequence constitutes a code that stores the genetic information of the cell (Figure 1).

### Torsional stress shapes DNA

**FIGURE 2:**
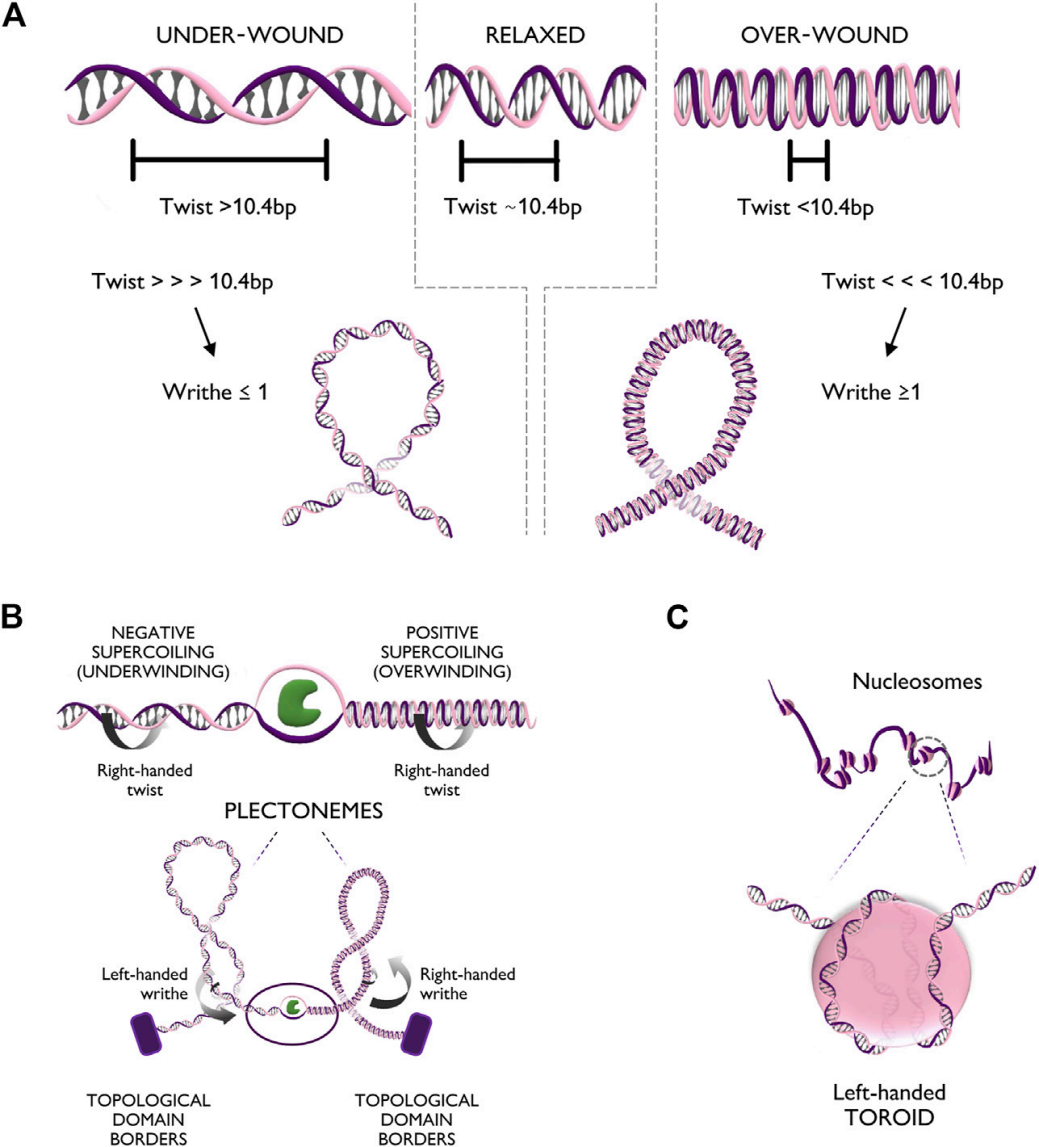
Torsional stress deforms the DNA fiber in multiple configurations. **(A)** Top panel: Different levels of twist of the DNA helix, respectively relaxed (bps per turn ∼10.4), under-wound (bps per turn >10.4), and over-wound (bps per turn <10.4). Bottom panel: Extreme twist levels result in further conformational changes in which the helix bends on itself generating positive writhe (right handedness) or negative writhe (left handedness). **(B)** Top panel: Unwinding of the DNA double helix creates positive supercoiling (over-winding) ahead of the unwinding factor (in green) and negative supercoiling (under-winding) behind. Bottom panel: Supercoiling can generate additional tridimensional rearrangements of the chromatin fiber such as plectonemes with positive or negative writhe. **(C)** Zoom-in of the DNA configuration of a nucleosome unit. The helix, characterized by right handedness, wraps around the nucleosome core particle in the form of a left handed spiral (toroid).

The DNA helix can assume different configurations, depending on the chemical environment surrounding it (Rich, 1993). In general, DNA assumes the most stable configuration, which is the one minimizing the free energy of the molecule. Any process that alters the bending or twisting of DNA increases its free energy, thus generating torsional stress. In a linear unconstrained molecule, torsional stress can be released simply by the rotation of one filament around the other. Nevertheless, in human chromatin, stress cannot be naturally relieved, due to the presence of DNA-binding proteins, such CTCF and histones, that form topological barriers (Gerasimova et al., 2016). Torsional stress can modify either the twist or the writhe of the helix (Figure 2A). The twist represents the degree of coiling of the double helix. In its most common conformation, the helix is right-handedly coiled around its axis with a frequency of one turn every ∼10.4 basepairs (Wang, 1979). If more right-handed rotation is applied, the frequency of turns increases (Tw < 10.4 bp) causing over-winding of the helix; *vice versa*, if left-handed rotation is applied, the frequency of turns decreases (Tw > 10.4 bp), causing under-winding of the helix. Over a critical value of twist, bending becomes more energetically favorable, leading to supercoiling of DNA. Supercoiling is characterized by the writhe value (Wr) as the number of times that the double helix crosses its central axis. The writhe can be positive (Wr ≥ 1) or negative (Wr ≤ −1), depending on whether supercoiling arises from excess in over-winding (Tw<<<10.4 bp) or under-winding (Tw>>>10.4 bp), respectively. *In vivo*, supercoiling can form complex tridimensional structures called plectonemes and toroids (Figures 2B, C). Plectonemes form during transcriptional elongation and consist of helix writhing around its longitudinal axis (Figure 2B), while toroids, typical of nucleosome units, form when the helix writhes around a cylinder (i.e., the histone core) into spirals (Figure 2C) (Jha et al., 2022).

### Topoisomerases can solve topological problems

Uncontrolled levels of torsional stress can give rise to topological problems as knots, catenates, and supercoils. Since these configurations can impede the accessibility of chromatin and the processivity of fundamental complexes, as the replicative and transcriptional ones, torsional stress should be handled by the cell. From bacteria to vertebrates, the proteins able to solve topological problems are called topoisomerases (see Table 1). Humans cells have six different topoisomerases which are subdivided into two categories: type 1 and type 2 (Pommier et al., 2016). Type 1 topoisomerases cut only one DNA strand, allowing its rotation around the intact one, thus acting as un-winders. Type 2 topoisomerases instead can cut both strands simultaneously, therefore relieving writhes.

**TABLE 1:** Main characteristics of human topoisomerases.

Topoisomerase	TOP1	TOP1 mt	TOP2A	TOP2B	TOP3A	TOP3B
**Type**	I	I	II	II	I	I
**Subtype**	B	B	A	A	A	A
**Localization**	nucleus	mitochondria	nucleus	nucleus	mitochondria	nucleus
			mitochondria	mitochondria		cytosol
**Substrate**	dsDNA	dsDNA	dsDNA	dsDNA	ssDNA	ssDNA and RNA
**Cofactors**	-	-	ATP, Mg^2+^	ATP, Mg^2+^	Mg^2+^	Mg^2+^
**Molecular weight**	100 kDa	70 kDa	170 kDa	180 kDa	112 kDa	98 kDa
**Genomic location**	20	8	17	3	17	22
**Supercoiling (Sc) target**	+ and -	+ and -	+ and -	+ and -	only -	only -
**Topological problem**	R loops	catenates	catenates	catenates	hemicatenates	catenates
	supercoils in replication forks		knots	knots	precatenates	knots
	supercoils in transcription		precatenates			R loops
			supercoils in replication forks			supercoils in transcription
**Biological process involved in**	replication	mitochondrial replication	replication	transcription	replication	transcription
	transcription	mitochondrial transcription	transcription	TADs organization	mitosis	heterochromatin formation
	mitosis	mitochondrial translation	heterochromatin formation	V(D)J recombination	heterochromatin formation	
**Effects upon loss/inhibition**	replication stalling	increased mitochondrial glycolysis	impaired DNA replication	decreased neuronal genes expression	sister chromatid exchanges	R-loops accumulation
	R-loops accumulation	impaired mitochondrial translation	impaired mitotic chromosome maintenance	defects in neural development	defective chromosome segregation	defective neuronal synapses
	genome instability	delayed tumorigenesis	impaired chromosome segregation	impaired B cell development	mitotic catastrophe	
**Dysfunctions correlated with**	Autism	Frequent SNPs	Autoimmunity syndrome lupus	Autism	Bloom syndrome	Carcinogenesis
	Aicardi-Goutieres syndrome		Cancer	B cell deficiency	Acute Myeloid Leukemia	Neurological disorders
	SCL70 autoimmune syndrome			Developmental delay	Mitochondrial diseases	Premature ageing

The six human topoisomerases have different functions, some specific and some partially redundant (see Table 1). While some topoisomerases are restricted to the mitochondria (TOP1mt and TOP3A) or can cut both DNA and RNA (TOP3B), three are important for the global organization of genomic DNA: TOP1, TOP2A, and TOP2B (Pommier et al., 2016).

Although the mechanism of cleavage and re-ligation of the different subtypes is now well characterized, much about topoisomerases remains to be discovered. Identifying their molecular partners and their mechanisms of target recognition is key to understanding how their activity is regulated. Elucidating these aspects would also advance cancer therapies, as many topoisomerase inhibitors were proved efficient to treat cancer (Table 2).

**TABLE 2:** Main human topoisomerase inhibitors.

Target	Class	Name	Origin	PubChem CID	Mechanism
**Type I**	Alkaloids	Camptothecin	from *Camptotheca acuminata*	24360	stabilizes cleavage complex
		Belotecan	camptothecin derivative	6456014	stabilizes cleavage complex
		Lurtotecan	camptothecin derivative	60956	stabilizes cleavage complex
		Topotecan	camptothecin derivative	60700	stabilizes cleavage complex
		Irinotecan (CPT-11)	camptothecin derivative	60838	stabilizes cleavage complex
		Exatecan	camptothecin derivative	151115	stabilizes cleavage complex
		Deruxtecan	exatecan derivative	118305111	stabilizes cleavage complex
		Nitidine	from *Zanthoxylum nitidum*	4501	stabilizes cleavage complex
		Topovale (ARC-111)	nitidine derivative	9888428	stabilizes cleavage complex
		Genz-644282	nitidine derivative	10294813	stabilizes cleavage complex
	Anthracyclines	Aclarubicin (Aclacinomycin A)	from S*treptomyces galilaeus*	451415	stabilizes cleavage complex
	Indenoisoquinolines	Indimitecan	synthesis	11519397	stabilizes cleavage complex
		Indotecan	synthesis	10294813	stabilizes cleavage complex
	Benzochromenones	β-Lapachone	from *Tabebuia avellanedae*	3885	catalytic inhibitor
	Triterpenoids	Betulinic acid	from *Betula alba*	64971	catalytic inhibitor
**Type II**	Alkaloids	Ellipticine	from *Ochrosia elliptica*	3213	intercalating poison
	Acridines	Amsacrine (m-AMSA)	aminoacridine derivative	2179	intercalating poison
	Anthracenediones	Mitoxantrone	synthesis	4212	intercalating poison
	Anthracyclines	Daunorubicin (Daunomycin)	from *Streptomyces peucetius*	30323	intercalating poison
		Doxorubicin (Adriamycin)	daunorubicin derivative	31703	intercalating poison
		Epirubicin	daunorubicin derivative	41867	intercalating poison
		Idarubicin	daunorubicin derivative	42890	intercalating poison
		Aclarubicin (Aclacinomycin A)	from S*treptomyces galilaeus*	451415	catalytic inhibitor
	Anthracenyl peptides	Merbarone	thiobarbituric acid derivative	4990817	catalytic inhibitor
	2H-chromen-2-ones	BNS-22	GUT-70 derivative	25265819	catalytic inhibitor
	Bisdioxopiperazines	ICRF187 (Dexrazoxane)	dioxopiperazine derivative	71384	catalytic inhibitor
		ICRF-193	dioxopiperazine derivative	115150	catalytic inhibitor
	Coumarins	Novobiocin (Albamycin)	from *Streptomyces niveus*	54675769	catalytic inhibitor
	Epipodophyllotoxins	Etoposide (VP-16)	podophyllotoxin derivative	36462	stabilizes cleavage complex
		Teniposide (VM-26)	podophyllotoxin derivative	452548	stabilizes cleavage complex
		F14512	podophyllotoxin derivative	25229664	stabilizes cleavage complex

## Main text

### Women’s contribution to the field of supercoiling and genome organization

Inspired by this Special Issue, here we review the research on supercoiling and genome organization, focusing exclusively on contributions of women as first authors.

#### The genome includes positive and negative supercoiled domains

In cells, both bacterial and eukaryotic, DNA is kept in a partially negative supercoiled state (Giaever and Wang, 1988). Any process involving the binding of a protein to DNA generates torsional stress that alters the equilibrium of supercoiling. In particular, one of the most relevant processes for supercoiling formation is transcription. Its importance was first suggested in the “twin-supercoiled-domain” model, which describes how advancing polymerases would over-wind DNA downstream while under-winding it upstream, forming positive and negative supercoiled domains (Figure 2B) (Liu and Wang, 1987). The generation of positive and negative supercoiled domains was first shown in yeast plasmids almost 40 years ago (Giaever and Wang, 1988).

Like plasmids, human chromosomes can also form under-wound and over-wound domains, which exhibit differential chromatin compaction (Naughton et al., 2013). Underwound regions are generally decompacted in comparison to the more compacted overwound regions (Naughton et al., 2013). Accordingly, the same genomic distance between two loci appears bigger in underwound than in overwound domains, when measured by DNA-FISH (Naughton et al., 2013). Interestingly, the distance separating loci of underwound domains decreases upon treatment with bleomycin, a drug that introduces double stranded DNA breaks, allowing the dissipation of torsional stress (Naughton et al., 2013). This indicates that the cytological decondensation characterizing underwound domains depends on torsional stress.

Notably, it was proposed that supercoiling can generate not only under- and over-wound domains but also TADs. Although supercoiled domains do not exactly match TADs (Krassovsky et al., 2021), these partial discrepancies could arise both from different resolution limits of mapping techniques and from entanglements of distinct supercoiled domains, as shown already with *in vitro* studies (Yan et al., 2018).

#### Topoisomerase positioning contributes to chromatin organization

TADs borders are known to be enriched with CTCF and cohesin, with cohesin facing the inner side of the TAD (Sanborn et al., 2015; Fudenberg and Imakaev, 2016). Interestingly, around ∼50% of these cohesin/CTCF bound borders colocalize also with TOP2B, which specifically positions at the outer side of the CTCF border, opposite to cohesin (Uusküla-Reimand et al., 2016). Since CTCF borders are natural barriers to supercoiling dissipation, TOP2B positioning could be essential in regulating torsional stress and stabilizing TADs (Björkegren and Baranello, 2018).

Indeed, topoisomerase 2 (TOP2) is mostly depleted within transcribed regions, which are instead enriched in topoisomerase 1 (TOP1) (Naughton et al., 2013). The presence of TOP1 could help polymerases during elongation, by relieving twisting preferentially ahead of the fork, while the absence of TOP2 would favor the generation of plectoneme structures, hypothesized of constituting loops (Kim et al., 2022).

Raising the level of transcription is expected to increase the level of supercoiling generated and, consequently, the need for its regulation through topoisomerases. Accordingly, while moderately expressed genes recruit almost only TOP1, highly transcribed genes need the additional help of TOP2 to control the excess of supercoiling (Kouzine, et al., 2013). Also, while TOP1 is generally distributed over a broad region upstream of the promoter, TOP2 is focally recruited at the transcription start site (TSS). The preferential recruitment of TOP2 at TSSs of highly expressed genes might be associated with the need to resolve plectonemes accumulated at these sites.

#### Supercoiling and transcription affect chromatin condensation

Changes in transcriptional activity not only impacts the level of supercoiling but also have an effect on chromatin density. Transcription inhibition causes large-scale chromatin compaction, measured by a decreased distance between genomic loci that belong to underwound domains (Naughton et al., 2013). Upon drug washout and transcription restoration, compaction is reverted to decondensation. Compaction is also produced upon topoisomerases inhibition (both types 1 and 2). Nevertheless, when transcription and topoisomerases are simultaneously inhibited, the loci distance remains unaltered, thus it has been suggested that compaction observed upon transcription inhibition requires topoisomerase activity (Naughton et al., 2013).

In agreement with Naughton et al. (2013) findings, we found that transcription or topoisomerases inhibition leads to global chromatin compaction, measured by super-resolution imaging of DNA (Neguembor et al., 2021). Inhibition of topoisomerases also affects chromatin looping, impairing cohesin loading and extrusion. Conversely, when cohesin overloading is experimentally forced by depletion of its loader WAPL, chromatin appears more homogeneous or “blended”, with decreased segregation of heterochromatin and euchromatin (Neguembor et al., 2021). Moreover, strongly blended chromatin accumulates negative supercoiling (Neguembor et al., 2021). Interestingly, WAPL deficient cells were shown to have more and longer chromatin loops (Gassler et al., 2017; Haarhuis et al., 2017; Wutz et al., 2017). Altogether, these findings suggest that cohesin could limit supercoiling dissipation, as previously reported *in vitro* for the bacterial looping protein LacI (Yan et al., 2018). Of note, modelling data show that the friction existing between cohesin and chromatin, due to the presence of nucleosomes and DNA-binding proteins, can control supercoiling levels (Rusková and Račko, 2021). Supercoiling, even at low levels, could act as a force driving loop extrusion *via* chemical potential. In fact, the incorporation of relaxed chromatin inside the supercoiled loop temporarily drops down its energy, towards a more energetically favorable state (Rusková and Račko, 2021). Indeed, *in vitro* studies agree with this model, since they show how the accumulation of supercoiling decreases the variation in looping probability, shifting the equilibrium completely to the looped state (Yan et al., 2021).

#### Supercoiling and transcription are mutually regulated

The studies discussed so far have revealed how the level of supercoiling alters the density and topology of the chromatin fiber. Nevertheless, even though the generation of supercoiling through transcription has been studied extensively, only recently has research focused on the possible impact of supercoiling on transcription itself.

The model from Liu and Wang (1987) drew a first hypothesis on how multiple transcriptional units would modulate supercoiled domains. Supercoiling would be annihilated between two polymerases moving synchronously in the same direction, and accumulate in the case of convergent or divergent polymerases, generating a positive or negative supercoiled domain between them, respectively (Liu and Wang, 1987).

To study how this process would happen *in vivo*, in the context of multiple transcribing polymerases, Heberling et al. (2016) constructed two models, one stochastic and one “torque-assisted”, for transcriptional elongation of three RNA polymerases proceeding in the same direction. In the stochastic model, the mean translocation rate, pause frequency, and duration of polymerases are constant and chosen *a priori*. Instead, in the torque-assisted model, these parameters are dynamically updated depending on the amount of torsion generated between each polymerase and its neighboring polymerases. Indeed, average transcription time gets 37.5% shorter in the torque-assisted model than in the stochastic one, providing a mechanistic explanation of cooperative behavior of polymerases observed in *E. coli* (Epshtein and Nudler, 2003). A similar model based on live-imaging in human cells has further corroborated the idea that torsional stress could coordinate polymerase activity by tuning their speed and pausing (Tantale et al., 2016).

In bacteria, supercoiling has been proven to be the primary source of transcriptional bursting during active transcription (Chong et al., 2014). *In vivo* findings in *E. coli* from Kim et al. (2019) confirmed the previously mentioned models and added the observation that polymerases can influence their dynamics through transcription-induced supercoiling even at remarkably long distances (>2 kb), spanning different genes. In line with this, computational simulations revealed that supercoiling can mediate transcription at multiple length scales (Geng et al., 2022). At a single-gene scale, supercoiling brings to the collective motion of co-transcribing RNA polymerase molecules, while at a multi-gene scale, supercoiling can mediate regulation of the transcription kinetics of adjacent genes. Indeed, in *E. coli*, transient negative supercoiling generated by transcription can activate the divergently coupled supercoiling-sensitive Leu-500 promoter, proportionally to the level of transcription and length of the transcripts (Zhi et al., 2017).

#### Cutting-edge sequencing and imaging approaches provide new insights into genome organization

The interest in how the genome organizes in the three-dimensional space has grown in the last decade. The major impulse behind this investigation is the idea that chromatin structure can directly influence cell function and identity. The need to delve deeper into this topic has stimulated the development of new cutting-edge approaches based on sequencing and on imaging to study genome organization (reviewed in Lakadamyali and Cosma, 2020; Jerkovic´ and Cavalli, 2021; Jung and Kim, 2021).

Among the sequencing-based approaches, techniques have constantly evolved to decrease the number of cells required and increase the genomic resolution. Also, new methods have been developed to overcome the limitation of most proximity-ligation methods that can only detect pair-wise interactions as they rely on the physical ligation of neighbouring fragments-ends. Recent techniques, such as SPRITE (Split-Pool Recognition of Interactions by Tag Extension) and ChIA-Drop (Chromatin Interaction Analysis *via* Droplet-based and barcode-linked sequencing), can detect multiway interactions by performing a physical separation of interacting domains (in wells or in droplets, respectively) followed by pooled-tagging and sequencing (Quinodoz et al., 2018; Zheng et al., 2019). The identification of multi-way interactions and complex contacts will be particularly relevant to delve deeper into chromatin topology and supercoiling.

Even in the imaging field, many advances have been recently made to expand our research tools. For high-resolution distance mapping, Mateo et al. (2019) developed ORCA (Optical Reconstruction of Chromatin Architecture), an optical method that allows tracing of DNA in 2 kb steps in single cells. To study genome dynamics with high spatiotemporal resolution, Clow et al. (2022) optimized the Casilio (CRISPR-Cas9-Pumilio) system (Cheng et al., 2016) for the simultaneous imaging of multiple non-repetitive DNA sequences in live cells, exploiting defective Cas9 and engineered sgRNAs recognized by fluorescently-tagged proteins. To probe proximity at specific genomic loci, Mota et al. (2022) recently developed FRET-FISH (Fluorescence Resonance Energy Transfer combined with DNA Fluorescence *In Situ* Hybridization), a method that could be applied in the future to study the formation of loops and condensates in single cells. Finally, a new method called “Modelling immuno-OligoSTORM” (MiOS), allows modelling of gene folding at nucleosome resolution by combining Oligopaint, DNA-PAINT (DNA Points Accumulation for Imaging in Nanoscale Topography), Hi-C (High-throughput sequencing Chromosome conformation capture), MNase-seq (Micrococcal Nuclease digestion with deep sequencing) and computational modelling (Neguembor, Arcon, Buitrago et al., 2022). Through MiOS, we were able to observe how pluripotency genes fold differently in pluripotent *versus* somatic cells.

## Discussion

Further development of new techniques will hopefully contribute to addressing key open questions in the supercoiling field. One of the most crucial issues regards the specific mechanism of action of different topoisomerases. Evidence shows that TOP1, TOP2A, and TOP2B can all relieve both negative and positive supercoiling. Nevertheless, it is not clear if different topoisomerases have preferential biases towards the relief of a type of supercoiling *versus* the other. Also, topoisomerases seem to be differentially positioned with respect to TADs and Polymerase II, possibly because of variation in specific protein-binding domains. It would be extremely interesting to investigate which factors are regulating their activities and what is the mechanism determining the basal state of genomic negative supercoiling. Moreover, the temporal dynamics of supercoiling *in vivo* are yet to be investigated, but the invention of ways to study supercoiling in living cells could eventually fill this gap. Finally, since transcription-associated supercoiling can contribute to genomic instability, a better understanding of topological stress regulation in human cells will be fundamental for cancer therapy, particularly in the context of oncogenic translocations (Gothe et al., 2019).

Despite the great progress in the study of genome architecture, there is still a tremendous lack of techniques for the study of supercoiling in eukaryotes. The only methods available have been developed just in the last few years and are the following two: the use of biotinylated trimethylpsoralen (bTMP), which preferentially intercalates in negatively supercoiled regions (Naughton et al., 2013), and the chromatin immunoprecipitation of GapR (GapR-seq), a bacterial protein which preferentially binds positively supercoiled regions (Guo et al., 2021). As a result, while the existence of differentially supercoiled domains in yeast plasmids –not chromosomes- was demonstrated already in 1988 (Giaever and Wang, 1988), it took another 25 years to show it in human cell chromatin (Naughton et al., 2013).

We would like to highlight that the discovery of supercoiled domains, together with all the ones mentioned in the main body of this article, were made by women listed as first authors in the publications. In the spirit of this Special Issue, we decided to focus on their work since we believe that they have directly contributed to a new emergent perspective of supercoiling and topoisomerases as key-players in genome organization. Still, in the field of genome organization only 25% of the 50 most cited articles includes women listed as first authors and only 16% have women listed as last authors. Again, the same disparity emerges in the sub-fields of supercoiling (33.3% female first authors and 36% female last authors), topoisomerases (34% and 6%, respectively) and transcription (35.4% and 10.2%, respectively)^1^. It is no secret that women still suffer almost worldwide from a dramatic gender gap in academia, in terms of salary, rate of pay increase, and probability of holding tenure-track positions (Ding et al., 2021). Nonetheless, this gap extends beyond salary and job position, affecting also the recognition in publications. In the scientific field, women are found to be much less likely credited as authors than men, both for articles and patents (Ross et al., 2022; Liu et al., 2023). Furthermore, among scientific disciplines, Biology displays the biggest disparity in terms of number of citations and productivity per author (Huang et al., 2020). While part of this gap is directly ascribed to differences in the publishing careers and dropout rates, still the reasons behind citations disparity has not been completely uncovered (Huang et al., 2020). On this regard, a meta-analysis of life sciences literature found a correlation between the impact of publications, their authors’ gender, and the use of “positive” words for describing scientific results, as “novel”, “unique”, and “promising” (Lerchenmueller et al., 2019). The analysis highlights how male authors tend to present their research more favourably than women do, and how this difference, particularly evident in high-impact journals, reflects in the number of downstream citations. Indeed, differences in language style related to gender had already been reported decades ago, indicating that women employ a more prominent use of “powerless” linguistic features, such as hesitations, disclaimers and hedges, while men instead tend to speak more assertively (Lakoff, 1975). However, this observation has been further reformulated, proposing a relation of language style to the social power of the communicator, more than to the gender *per se* (O’Barr, 1982). Yet, whether the difference arises from inner natural sex-related characteristics or from patriarchal societal conditioning, the linguistic style used by women could negatively impact public perception of their published work, in terms of competence and credibility (Blankenship and Holtgraves, 2005). Nevertheless, a hedging language is necessary in science, when used to discriminate theories and models from facts. A more tentative style could lead to more cautious conclusions than assertive ones, even starting from the very same experimental results. This ultimately might influence the evolution and popularity of specific gender-driven models within the scientific community. Thus, we raise the question if it is desirable that women should empower their scientific communication, or rather that men should be more careful in using assertive and self-promoting statements, for the sake of accurate science. We personally believe that progress on both sides is important to overcome gender bias in scientific publications and their impact.

We hope that raising awareness of the contribution of women to the fields of 3D genome organization and supercoiling could help fighting gender gaps and most importantly could encourage women to participate in research with the confidence and power to make their brilliant and innovative ideas reachable to the scientific community.

## Data Availability

The original contributions presented in the study are included in the article, further inquiries can be directed to the corresponding authors.

## Footnotes

1 These data were collected by using the software Publish or Perish 8.8.4275.8412 on the 31st of January, 2023. Search was conducted in the Google Scholar database setting at 100 the maximum number of results, which were filtered for the year of publication (from 2013 to 2022) and keywords (“genome organization”, “supercoiling”, “topoisomerases”, and “transcription”). The first 50 papers were ranked according to the number of cites per year, and gender was assigned to the first/last author upon bibliographic research. In the cases of single authors, these were considered last authors.
